# Factors That Influence Pancreatic Beta Cell Function and Insulin Resistance in Newly Diagnosed Type 2 Diabetes Patients: A Sub-Analysis of the MARCH Trial

**DOI:** 10.1007/s13300-018-0393-5

**Published:** 2018-03-09

**Authors:** Yan Duan, Jia Liu, Yuan Xu, Ning Yang, Wenying Yang, Guang Wang

**Affiliations:** 1 Department of Endocrinology, Beijing Chao-Yang Hospital, Capital Medical University, Beijing, 100020 People’s Republic of China; 2 Department of Endocrinology, China-Japan Friendship Hospital, Beijing, 100029 People’s Republic of China

**Keywords:** Acarbose, Beta cell function, Diabetes, HOMA-β, HOMA-IR, Insulin resistance

## Abstract

**Introduction:**

The Metformin and Acarbose in Chinese as the initial Hypoglycemic treatment (MARCH) trial has demonstrated a similar efficacy in HbA1c reduction between acarbose and metformin treatments in newly diagnosed type 2 diabetes mellitus (T2DM) patients. The current sub-analysis of the MARCH trail aims to evaluate the baseline characteristics that may influence the improvement of pancreatic β-cell function and insulin resistance after acarbose therapy in Chinese patients with newly diagnosed T2DM.

**Methods:**

Of the 784 patients who entered the MARCH trail, 391 were assigned to the acarbose therapy group; 304 of these completed 48 weeks of follow-up of acarbose therapy. At 48 weeks, on the basis of the tertiles of change in homeostasis model assessment–beta cell function (∆HOMA-β) and homeostasis model assessment–insulin resistance (∆HOMA-IR), the subjects were divided into lowly, mediumly, and highly improved groups.

**Results:**

In the highly improved HOMA-β group, patients had higher systolic blood pressure (SBP), 2-h postprandial blood glucose (PBG), hemoglobin A1c (HbA1c), and lower high-density lipoprotein cholesterol (HDL-c), fasting serum insulin (FINS) concentration, and HOMA-IR in comparison to the lowly improved group (*p* < 0.05). A positive correlation was observed between HbA1c, SBP, and highly improved ∆HOMA-β (*p* < 0.05), while an inverse correlation was evident between HDL-c and highly improved ∆HOMA-β (*p* < 0.05). The highly improved HOMA-IR group had a significantly higher body mass index (BMI), fasting blood glucose (FBG), FINS concentration, and HOMA-β in comparison to the lowly improved group (*p* < 0.05). A positive correlation was observed between FBG, waist circumference, and highly improved HOMA-IR (*p* < 0.05).

**Conclusion:**

Newly diagnosed T2DM Chinese patients with lower baseline HDL-c and higher HbA1c and SBP values are more likely to achieve improvement in beta cell function whereas baseline fasting blood glucose and waist circumference were the significant factors associated with improvement in insulin resistance with acarbose therapy.

**Trial Registration:**

The clinical trial registry number was ChiCTR-TRC-08000231.

## Introduction

Type 2 diabetes mellitus (T2DM) is a common chronic metabolic disease that has become a silent epidemic and a major global health burden over the years. China has the largest number of diabetic patients with a growing population of approximately 92.4 million [1, 2]. Metformin is the most commonly prescribed oral antidiabetic drug, taken by more than 150 million people annually [3]. The American Diabetes Association (ADA) and European Association for the Study of Diabetes (EASD), as well as many other authoritative clinical practice guidelines, have recommended metformin as the first-line therapy for the treatment of T2DM because of its hypoglycemic effect and long-term safety record [4, 5]. Besides metformin, alpha-glucosidase inhibitors (AGIs) are another class of ADA- and EASD-recommended oral hypoglycemic drugs, commonly prescribed in the East Asian population [6]. Several studies have reported postprandial hyperglycemia as a predominant contributor in newly diagnosed T2DM in China [7–9]. This may due to the high consumption of carbohydrate-rich white rice by the Chinese in comparison to other populations [10, 11]. Hence, acarbose is a widely prescribed hypoglycemic drug in China. The MARCH (Metformin and Acarbose in Chinese as the initial Hypoglycemic treatment) trial, conducted with 788 newly diagnosed Chinese type 2 diabetic patients, demonstrated a similar reduction of hemoglobin A1c (HbA1c) after 48 weeks of treatment with either acarbose or metformin [12]. However, the clinical features that impact the effectiveness of acarbose therapy in Chinese T2DM patients have not been previously studied. Hence, we used the data from the MARCH trial to determine the correlation between the baseline characteristics and the effectiveness of acarbose therapy by assessing the improvement in pancreatic beta cell functions and insulin resistance. The aim of the current study was to evaluate the baseline characteristics that may influence the improvement of pancreatic β-cell functions and insulin resistance after 48 weeks of acarbose therapy.

## Methods

### Design and Participants

This study is a sub-analysis of the MARCH trial, a randomized, open-label, non-inferiority trial (ChiCTR-TRC-08000231) that compared acarbose with metformin as an initial therapy in newly diagnosed T2DM patients. The enrollment criteria, baseline protocol, and diagnostic definitions have been reported previously [12].

Based on 1999 WHO diagnosis criteria, the study enrolled a total of 784 newly diagnosed T2DM patients, aged between 30 and 70 years, from 11 clinical sites. A total of 391 of the participants were assigned to the acarbose therapy group. The current study included 304 patients who completed follow-up at 48 weeks after acarbose therapy. Patients who had not received any oral antidiabetic drug or those who were previously treated for a short term and had discontinued 3 months before the enrollment were included in the study. Patients with a history of unstable angina, acute myocardial infarction, liver function impairment, renal function impairment, hematological diseases, chronic hypoxic diseases (emphysema and cor pulmonale), intestinal surgery, and infectious disease were excluded from the study.

### Measurements

The baseline measurements included assessment of the body weight, waist circumference, hip circumference, systolic blood pressure (SBP), diastolic blood pressure (DBP), oral glucose tolerance test [fasting blood glucose (FBG) and 2-h postprandial blood glucose (PBG)], fasting serum insulin (FINS), lipid profile [triglycerides (TG), total cholesterol (TC), low-density lipoprotein cholesterol (LDL-C), and high-density lipoprotein cholesterol (HDL-C)], and HbA1c.

Also, the total energy and the daily intake of carbohydrate, fat, protein, and fiber in the diet were recorded at baseline. Patients were followed up by anthropometric and laboratory index at 24 and 48 weeks.

Homeostasis model assessment of β-cell function (HOMA-β) and homeostasis model assessment of insulin resistance (HOMA-IR) were calculated by using the following equations: HOMA-β = 20 × FINS (mIU/L)/[FBG (mmol/L) − 3.5], HOMA-IR = [FBG (mmol/L) × FINS (mIU/L)/22.5] [13, 14].

The improvement of HOMA-β was evaluated using ∆HOMA-β: 48-week HOMA-β − baseline HOMA-β; and the improvement of HOMA-IR was evaluated using ∆HOMA-IR: baseline HOMA-IR − 48 weeks HOMA-IR.

### Distribution of Patients

At 48-week follow-up, the subjects were categorized into three groups, lowly improved (LI), mediumly improved (MI), and highly improved (HI), based on the tertiles of change noted in HOMA-β (∆HOMA-β) and HOMA-IR (∆HOMA-IR).

In relation to HOMA-β, the LI group had ∆HOMA-β <− 13.9, the MI group had − 13.9 ≤ ∆HOMA-β < 13.9, and the HI group had ∆HOMA-β ≥ 13.9. The LI group in ∆HOMA-IR was assessed at < 0.6, the MI group had 0.6 ≤ ∆HOMA-IR < 2.9, and the HI group had ≥ 2.9 resistance.

### Statistical Methods

SPSS version 21.0 was used to perform statistical analysis of the study. Continuous variables that had normal distributions were expressed as mean ± standard deviation (SD). Student *t* test and one-way ANOVA were adopted to analyze the differences in characteristics between the groups. Continuous variables that did not have normal distribution were expressed as median with a range of upper and lower quartiles and analyzed using a non-parametric test. Discontinuous variables were expressed as a percentage and analyzed using Chi-square test. In addition, logistic regression was performed to analyze the factors that may influence ∆HOMA-β and ∆HOMA-IR. Statistical significance was defined as *p* < 0.05.

### Compliance with Ethics Guidelines

All procedures performed in the study were in accordance with the ethical standards of the institutional and national research committee and with the 1964 Declaration of Helsinki and its later amendments or comparable ethical standards. Informed consent was obtained from all individual participants included in the study.

## Results

### Baseline Analysis of Different Improvements of HOMA-β

Out of the 784 enrolled patients, 304 patients on acarbose therapy completed the 48-week follow-up. The baseline characteristics of the different ∆HOMA-β groups are summarized in Table 1. A significant increase in baseline PBG (11.70 ± 2.89 vs 13.06 ± 2.54 vs 12.61 ± 2.88 mmol/l) and HbA1c (7.13 ± 0.97 vs 7.52 ± 1.10 vs 7.68 ± 1.32%) and a significant decrease in the HDL-c (1.27 ± 0.31 vs 1.21 ± 0.27 vs 1.18 ± 0.25 mmol/l) were noted among the three groups.

**Table 1:** Baseline characteristics of different ∆HOMA-β groups

Parameters	< − 13.9	− 13.9 ≤ ∆HOMA-β < 13.9	≥ 13.9	*p* value
	*n* = 101	*n* = 102	*n* = 101	
Age (years)	50.4 ± 9.0	50.4 ± 9.0	50.4 ± 9.8	0.982
Male % (*n*)	53.5 (54)	62.7 (64)	60.4 (61)	0.318
Weight (kg)	71.4 ± 10.6	69.6 ± 9.9	71.9 ± 9.7	0.744
BMI (kg/m^2^)	26.15 ± 2.50	25.35 ± 2.5	26.05 ± 2.31	0.774
Waist circumference (cm)	89.8 ± 8.3	89.2 ± 8.6	90.5 ± 7.6	0.528
Hip circumference (cm)	100.0 ± 7.6	98.3 ± 7.1	99.9 ± 7.5	0.944
SBP (mmHg)	121.7 ± 12.5	123.0 ± 12.7	125.2 ± 12.5^a^	0.050
DBP (mmHg)	80.1 ± 8.7	78.6 ± 9.2	79.4 ± 8.4	0.590
LDL-c (mmol/l)	3.10 ± 0.91	3.12 ± 0.84	3.16 ± 0.86	0.615
HDL-c (mmol/l)	1.27 ± 0.31	1.21 ± 0.27	1.18 ± 0.25^a^	0.020
TG (mmol/l)	1.71 (1.07–2.85)	1.91 (1.30–2.80)	1.84 (1.35–2.40)	0.370
TC (mmol/l)	5.29 ± 1.05	5.37 ± 1.19	5.14 ± 1.04	0.726
FBG (mmol/l)	7.88 ± 1.37	8.60 ± 1.38	8.22 ± 1.31	0.081
PBG (mmol/l)	11.70 ± 2.89	13.06 ± 2.54	12.61 ± 2.88^a^	0.021
HbA1c (%)	7.13 ± 0.97	7.52 ± 1.10	7.68 ± 1.32^a^	0.001
Ins (μIU/ml)	16.38 (11.17–21.97)	8.41 (5.11–13.47)	9.33 (5.67–13.02)^a^	< 0.001
HOMA-IR	5.82 (3.69–8.65)	3.13 (1.82–5.29)	3.32 (2.00–5.20)^a^	< 0.001
Hyperlipidemia % (*n*)	22.8 (23)	37.3 (38)	28.7 (29)	0.356
Fatty liver % (*n*)	0 (0)	1.0 (1)	5.0 (5)	0.012
TtEn (cal)	1359 (1145–1676)	1550 (1209–1985)	1409 (1168–1697)	0.859
TtCHO (cal)	187.0 (144.7–243.1)	201.6 (154.4–270.0)	199.9 (148.3–255.3)	0.206
TtFib (cal)	7.39 (4.87–9.54)	7.56 (5.13–11.35)	7.49 (4.77–13.40)	0.635
TtPro (cal)	47.43 (35.87–60.98)	58.32 (41.30–70.30)	49.38 (39.19–63.56)	0.979
TtFat (cal)	46.18 (35.23–62.46)	45.99 (35.49–68.12)	46.66 (35.55–59.83)	0.224

*BMI* body mass index, *SBP* systolic blood pressure, *DBP* diastolic blood pressure, *TC* total cholesterol, *LDL-C* low-density lipoprotein cholesterol, *HDL-C* high-density lipoprotein cholesterol, *TG* triglyceride, *FBG* fasting blood glucose, *PBG* 2-h postprandial blood glucose, *FINS* fasting insulin, *HbA1c* hemoglobin A1c, *HOMA-IR* homeostasis model assessment of insulin resistance, *HOMA-β* homeostasis model assessment of β-cell function, *TtEn* total energy, *TtCHO* total carbohydrate, *TtFib* total fiber, *TtPro* total protein, *TtFat* total fat, *Hyperlipidemia* history of hyperlipidemia, *Fatty liver* history of fatty liver

^a^Statistically significant differences compared to HOMA-β < − 13.9 group (*p* < 0.05)

In comparison to the LI group, the HI HOMA-β group had higher levels of SBP (121.7 ± 12.5 vs 125.2 ± 12.5 mmHg), lower fasting insulin (16.38 vs 9.33 μIU/ml) and HOMA -IR (5.82 vs 3.32) at baseline, and the differences were statistically significant (*p* < 0.05). The other parameters including age, BMI, and LDL-c had no significant differences among three groups at baseline. Also, the total energy and the daily intake of carbohydrate, fat, protein, and fiber in the diet at baseline had no significant differences among the groups.

### Factors Associated with Pancreatic β-Cell Function

The multiple logistic regression analysis showed that baseline HbA1c (OR 1.542; 95% CI 1.181–2.013), SBP (OR 1.026; 95% CI 1.001–1.050), and HDL-c (OR 0.310; 95% CI 0.100–0.957) were associated with HI HOMA-β and were statistically significant (*p* < 0.05) in comparison to LI HOMA-β (Table 2).

**Table 2:** Logistic regression analysis of the factors associated with ∆HOMA-β

	*p* value	OR	95% CI	
∆HOMA-β ≥ 13.9				
Sex = male	0.602	1.192	0.617	2.302
Age (years)	0.780	1.005	0.972	1.039
Waist circumference (cm)	0.631	0.990	0.952	1.030
HbA1c (%)	0.001	1.542	1.181	2.013
SBP (mmHg)	0.038	1.026	1.001	1.050
HDL-c (mmol/l)	0.042	0.310	0.100	0.957

Compared to ∆HOMA-β < − 13.9. Variables included in the model were male, age, baseline waist circumference

*HbA1c* systolic blood pressure, *HDL* cholesterol, *HbA1c* hemoglobin A1c, *SBP* systolic blood pressure, *HDL-C* high-density lipoprotein cholesterol

### Baseline Analysis of Different Improvement of HOMA-IR

The baseline characteristics of the different ∆HOMA-IR groups are summarized in Table 3. A significantly increasing trend in BMI (25.45 ± 2.32 vs 25.74 ± 2.40 vs 26.36 ± 2.61 kg/m^2^) and FBG (7.90 ± 1.32 vs 8.08 ± 1.27 vs 8.72 ± 1.42 mmol/l) was noted among all the groups. The HI group had a higher concentration of fasting insulin level (18.73 vs 7.53 μIU/ml) and HOMA-β (81.19 vs 35.63) in comparison to the LI group and the difference was statistically significant (*p* < 0.05). The other parameters like age, SBP, HbA1c and LDL-c did not have any significant differences among the groups. There were no significant differences in dietary intake of carbohydrate, fat, protein, and fiber at baseline among all the groups.

**Table 3:** Baseline characteristics of different ∆HOMA-IR groups

Parameters	< 0.6	0.6 ≤ ∆HOMA-IR < 2.9	≥ 2.9	*p* value
	*n* = 101	*n* = 102	*n* = 101	
Age (years)	50.5 ± 9.9	50.4 ± 8.7	50.3 ± 9.2	0.958
Male % (*n*)	61.4 (62)	57.8 (59)	57.4 (58)	0.568
Weight (kg)	70.3 ± 9.7	70.1 ± 9.1	72.6 ± 11.1	0.104
BMI (kg/m^2^)	25.45 ± 2.32	25.74 ± 2.40	26.36 ± 2.61^a^	0.009
Waist circumference (cm)	89.5 ± 7.6	88.6 ± 8.6	91.4 ± 8.1	0.097
Hip circumference (cm)	98.8 ± 6.6	99.0 ± 7.5	100.4 ± 8.0	0.131
SBP (mmHg)	123.3 ± 10.9	123.3 ± 14.7	123.2 ± 12.2	0.947
DBP (mmHg)	78.4 ± 8.1	79.6 ± 9.7	80.1 ± 8.3	0.167
LDL-c (mmol/l)	3.20 ± 0.74	3.06 ± 0.90	3.13 ± 0.96	0.332
HDL-c (mmol/l)	1.18 ± 0.26	1.25 ± 0.29	1.24 ± 0.29	0.277
TG (mmol/l)	1.78 (1.20–2.48)	1.78 (1.28–2.45)	2.03 (1.32–2.85)	0.389
TC (mmol/l)	5.18 ± 0.98	5.22 ± 1.19	5.40 ± 1.11	0.639
FBG (mmol/l)	7.90 ± 1.32	8.08 ± 1.27	8.72 ± 1.42^a^	< 0.001
PBG (mmol/l)	12.04 ± 2.81	12.54 ± 2.48	12.80 ± 3.13	0.058
HbA1c (%)	7.40 ± 1.18	7.41 ± 1.11	7.52 ± 1.19	0.429
Ins (μIU/ml)	7.53 (4.61–10.78)	9.34 (7.23–11.87)	18.73 (15.36–24.71)^a^	< 0.001
HOMA-β	35.63 (20.05–50.99)	44.75 (28.64–62.08)	81.19 (52.22–108.58)^a^	< 0.001
Hyperlipidemia % (*n*)	27.7 (28)	32.4 (33)	28.7 (29)	0.878
Fatty liver % (*n*)	2.0 (2)	2.9 (3)	1.0 (1)	0.614
TtEn (cal)	1510 (1188–1778)	1550 (1289–1966)	1611 (1229–1895)	0.657
TtCHO (cal)	206.9 (171.1–252.8)	220.0 (176.8–268.7)	229.7 (169.5–284.0)	0.107
TtFib (cal)	8.05 (4.82–10.86)	7.88 (5.95–11.15)	8.65 (5.34–11.89)	0.556
TtPro (cal)	52.32 (36.11–69.65)	55.27 (41.58–70.03)	55.42 (38.55–71.52)	0.687
TtFat (cal)	48.42 (35.69–64.93)	48.66 (34.51–63.99)	48.32 (35.17–63.46)	0.883

*BMI* body mass index, *SBP* systolic blood pressure, *DBP* diastolic blood pressure, *TC* total cholesterol, *LDL-C* low-density lipoprotein cholesterol, *HDL-C* high-density lipoprotein cholesterol, *TG* triglyceride, *FBG* fasting blood glucose, *PBG* 2-h postprandial blood glucose, FINS: fasting insulin, *HbA1c* hemoglobin A1c, *HOMA-IR* homeostasis model assessment of insulin resistance, *HOMA-β* homeostasis model assessment of β-cell function,* TtEn* total energy, *TtCHO* total carbohydrate, *TtFib* total fiber, *TtPro* total protein, *TtFat* total fat, *Hyperlipidemia* history of hyperlipidemia, *Fatty liver* history of fatty liver

^a^Statistically significant differences compared to HOMA-IR < 0.6 group (*p* < 0.05)

### Factors Associated with Insulin Resistance

According to the multiple logistic regression analysis, baseline FBG (OR 1.612; 95% CI 1.286–2.021) and waist circumference (OR 1.055; 95% CI 1.013–1.099), especially in the male population (OR 0.498; 95% CI 0.254–0.977), were associated with HI HOMA-IR and these differences were statistically significant in comparison to LI HOMA-IR (*p* < 0.05) (Table 4).

**Table 4:** Logistic regression analysis of the factors associated with ∆HOMA-IR

	*p* value	OR	95% CI	
∆HOMA-IR ≥ 2.9				
Sex = male	0.042	0.498	0.254	0.977
Age (years)	0.757	0.995	0.963	1.028
Waist circumference (cm)	0.010	1.055	1.013	1.099
FBG (mmol/l)	0.000	1.612	1.286	2.021
SBP (mmHg)	0.650	0.995	0.971	1.018
Total cholesterol (mmol/l)	0.088	1.004	0.999	1.008

Compared to ∆HOMA-IR < 0.6. Variables included in the model were male, age, baseline waist circumference, fasting blood glucose, systolic blood pressure, total-cholesterol

*FBG* fasting blood glucose, *SBP* systolic blood pressure

## Discussion

The MARCH trial demonstrated a similar efficacy in reducing the HbA1c with both acarbose and metformin as first-line therapy. However, the efficacy of acarbose in reducing 2-h postprandial glucose was greater than that of metformin. Similar results were reported by Rosenstock et al. [15] and Wu et al. [16].

Besides HbA1c reduction, the improvement in β-cell function and insulin resistance are the other important indicators for evaluating the efficacy of antidiabetic drugs in T2DM patients. However, the factors that influence the improvement of pancreas function and insulin resistance in the Chinese T2DM patients under acarbose therapy are still unclear. In this subgroup analysis of the MARCH study, we analyzed the baseline characteristics that improve the pancreatic β-function and insulin resistance, which may guide the selection of antidiabetic agents in patients with newly diagnosed T2DM.

In the current study, patients with HI HOMA-β had higher postprandial glucose and HbA1c and a lower fasting insulin and HOMA-IR. These results signify higher HbA1c and lower insulin resistant condition at baseline as favorable factors associated with significant improvement of pancreas β-cell function after acarbose therapy, in newly diagnosed Chinese T2DM patients. Chen et al. study reported a significant improvement in β-cell function with add-on acarbose therapy in patients previously treated with metformin [17]. Sun et al. reported a slight nonsignificant improvement in HOMA-β (66.8±41.3 vs 85.9±74.6) after 24 weeks of acarbose therapy [18].

In the present study, it was observed that the amelioration of HOMA-β had a positive correlation with baseline SBP and inverse correlation with baseline HDL-c. It is well documented that both decreased HDL-c and increased SBP are proven risk factors of arteriosclerosis [19, 20]. Studies in both prediabetic and diabetic patients have demonstrated a reduction in cardiovascular events following acarbose therapy with beneficial effects on a broad spectrum of CV risk factors [21, 22]. The STOP NIDDM study revealed a 49% and 34% relative risk reduction in the development of cardiovascular events and hypertension in prediabetic patients treated with acarbose [21].

For this study, SBP at baseline was significantly higher in the HI group of β-cell function than the other groups. The relationship between hypertension and the islet function is still not very clear. Previous studies have reported that elevated blood pressure is related to the secretion of insulin and increasing the prevalence of insulin resistance [23, 24]. Whereas, high SBP level but low HOMA-IR was found in the HI group, which may be explained by the inhibition of pancreatic β-cell function and insulin secretion influenced by the toxicity of high glucose in the HI group [25]. Previous animal experiments and clinical research have found improvement of pancreas function and hyperinsulinemia in hypertensive subjects treated with acarbose [26–28]. Similar to that evidence, our study suggested that patients with elevated SBP may benefit from acarbose therapy by improving pancreas β-cell function. However, we did not find a correlation between the baseline SBP and insulin resistance amelioration. Acarbose also positively influences lipid management in patients with T2DM, by decreasing LDL-c and triglyceride level and increasing HDL-c level [29, 30]. Our study suggests that low level of HDL-c and high level of SBP at baseline are associated with an improved β-cell function in patients treated with acarbose.

In the present study, it was observed that improved insulin resistance was significantly associated with higher baseline BMI, FBG, fasting insulin, and HOMA-β. In addition, a positive association between waist circumference and high improvement in insulin resistance was noted. This is inconsistent with the study conducted by Delgado et al. where a significant improvement in postprandial plasma glucose and insulin secretion and 15% reduction in insulin resistance were detected in obese patients treated with acarbose (*p* < 0.05) [31]. Furthermore, Rachmani et al. reported a reduction in insulin resistance and triglycerides following acarbose therapy in obese hypertensive patients with normal glucose tolerance [28].

Unlike other studies that compared the efficacy of acarbose and placebo in obese patients, our study analyzes the impact of baseline characteristics on the improvement of HOMA-IR and reveals that patients who have high BMI (average 26.36 ± 2.61 kg/m^2^) are likely to have significant improvement of insulin resistance. On the other hand, we do not find an association between BMI and waist circumference with HOMA-β improvement.

## Conclusion

Baseline HbA1c, HDL-c, and SBP influenced the outcome of beta cell function whereas waist circumference and FBG at baseline influenced insulin resistance in Chinese patients with newly diagnosed T2DM treated with acarbose. As patient-centered management is recommended in T2DM, it is advisable to analyze the baseline characteristics of patients and their changes during the treatment period, which may lead to best treatment options for individual patients.

### Limitation

This was a secondary analysis performed using the database from a previous clinical trial (i.e., MARCH). Hence, a prospective study with a larger sample size is necessary to confirm our results.
